# The predictive value of neutrophil to lymphocyte ratio on 30-day outcomes in spontaneous intracerebral hemorrhage patients after surgical treatment: A retrospective analysis of 128 patients

**DOI:** 10.3389/fneur.2022.963397

**Published:** 2022-08-22

**Authors:** Yiqin Zhao, Yanfeng Xie, Shengjie Li, Mingliang Hu

**Affiliations:** ^1^The First Clinical Medical School, Chongqing Medical University, Chongqing, China; ^2^Department of Neurosurgery, The First Affiliated Hospital of Chongqing Medical University, Chongqing, China; ^3^Department of Neurosurgery, Dianjiang People's Hospital of Chongqing, Chongqing, China

**Keywords:** neutrophil-to-lymphocyte ratio, spontaneous intracerebral hemorrhage, craniotomy, minimally invasive puncture and drainage, prognosis

## Abstract

**Objective:**

The purpose of this study was to explore the predictive value of the neutrophil-to-lymphocyte ratio (NLR) on 30-day outcomes in patients with spontaneous intracerebral hemorrhage (ICH) after surgical treatment.

**Methods:**

This retrospective study utilized data from patients with ICH who underwent craniotomy or minimally invasive puncture and drainage (MIPD) between January 2015 and June 2021. The patients meeting the inclusion criteria were divided into two groups according to 30-day outcomes, namely, the favorable outcome group and the poor outcome group. Sex, age, time from onset to admission, vital signs at admission, admission Glasgow Coma Scale (GCS) score, diabetes mellitus, hypertension, hematoma volume, hematoma location, surgical approach, and NLR at different time points were all recorded and analyzed.

**Results:**

A total of 128 patients were finally enrolled in this study, including 32 and 96 patients in the favorable outcome group and the poor outcome group, respectively. During the course of ICH, the changing trend of NLR was to increase first and then decrease and peaked within 48 h after surgery. In the univariate analysis, systolic blood pressure, admission GCS score, hematoma volume, surgical approach, and NLR within 48 h after surgery were statistically significant. In the multivariable analysis, NLR within 48 h after surgery (odds ratio [*OR*] = 1.342, *p* < 0.001) was an independent risk factor of the 30-day outcomes in patients with ICH after surgical treatment. The receiver operating characteristic (ROC) analysis showed that the best predictive cut-off value for NLR within 48 h after surgery was 12.35 [sensitivity 82.9%, specificity 81.8%, and area under the curve (AUC) 0.877] and 14.46 (sensitivity 55.1%, specificity 87.5%, and area under the curve 0.731) for the MIPD group and the craniotomy group, respectively.

**Conclusions:**

In the process of ICH, the value of NLR was increased first and then decreased and peaked within 48 h after surgery. NLR within 48 h after surgery was an independent risk factor of the 30-day outcomes in patients with ICH. The peak NLR >12.35 or 14.46 in patients receiving MIPD or craniotomy reflected a poor prognosis, respectively.

## Introduction

Spontaneous (non-traumatic) intracranial hemorrhage (ICH) is a fatal illness with a high risk of morbidity and mortality of global importance, which affects approximately 2 million people in the world each year (1). Some of these patients require timely surgical management and the prognosis of patients with ICH after a surgery is of great concern for both neurosurgeons and patients. However, there are several different shortcomings in the current prognostic indicators for patients with ICH who have received surgery (2).

The neutrophil-to-lymphocyte ratio (NLR) has emerged as a potential and readily available inflammation indicator and has played an increasingly important role in many clinical contexts, such as glioma, infective endocarditis, colorectal cancer, and acute ischemic stroke (3–6). Since the inflammatory response is involved in the secondary brain injury following ICH (7), NLR, a marker of inflammation, may be an indicator of prognosis for patients with ICH. Recent evidence suggested that NLR is a more convincing indicator of inflammation than other markers (8) and is closely associated with 30 and 90-day outcomes in patients with ICH (9, 10). However, as an important part of treatment, surgery was not considered in these studies. Surgical treatment may prevent hematoma expansion, block the release of inflammatory products from hematomas, and thus intervene in the pathological processes after disease onset (11). Several studies reported that NLR was independently associated with a 90-day poor outcome and 30-day mortality of patients with ICH after hematoma evacuation (12, 13). However, these studies neglected the severity of the brain tissue damage caused by different surgical. The prognostic value and trend of NLR in different surgical methods are also not clear.

The aim of this study was to analyze the variation rule of NLR and evaluate the relationship between NLR and short-term prognosis of patients with ICH undergoing craniotomy and minimally invasive puncture and drainage (MIPD).

## Materials and methods

This study was approved by the Medical Ethics Review Committee of the First Affiliated Hospital of Chongqing Medical University and the Dianjiang People's Hospital of Chongqing. All participants provided written informed consent. The work has been reported in line with the Standards for Reporting of Diagnostic Accuracy Studies (STARD) criteria.

### Patient selection

This current retrospective study collected data from 128 patients with ICH treated with craniotomy or minimally invasive surgery at the First Affiliated Hospital of Chongqing Medical University and the Dianjiang People's Hospital of Chongqing from January 2015 to June 2021. Among them, 83 patients were from the First Affiliated Hospital of Chongqing Medical University and 45 patients were from the Dianjiang People's Hospital of Chongqing.

Inclusion criteria: (1) Patients over the age of 18 years; (2) patients diagnosed with spontaneous ICH confirmed by computed tomography (CT) scan within 24 h from symptoms ictus; and (3) patients who underwent either craniotomy or MIPD within 24 h of admission.

Exclusion criteria: (1) Patients who had an infection for 2 weeks; (2) patients who had comorbidity that might affect the value of NLR, such as cancer, autoimmune disease, hematological disease, chronic heart disease, and renal or liver diseases; (3) patients who had been treated with immunomodulatory treatments; (4) patients who had been evaluated as Glasgow Coma Scale (GCS) score ≤ 5 at admission, or disability before disease onset; (5) patients who had incomplete follow-up data; and (6) patients who had the dissatisfied effect of the evacuation of hematoma or received re-operation.

### Preoperative management

All patients enrolled in the study were treated according to the guidelines of management from the American Heart Association/American Stroke and were divided into the craniotomy group and the MIPD group. When the patients arrived at the hospital, emergency CT was performed immediately to evaluate the size and location of the hematoma, diagnosing the level of consciousness was performed using GCS, and peripheral blood was collected for examination. The neurosurgical procedure for each patient was customized by experienced neurosurgeons based on the clinical conditions of patients with ICH at admission, including entry point, surgical approach, and bone window size.

### Surgery

#### Craniotomy group

The patients in the craniotomy group underwent conventional craniotomy operations to clear the hematoma. The location of the bone window was determined according to the site of hematoma reflected by brain CT of the patient's preoperative condition. Once entering the hematoma cavity, suction and bipolar cautery were used to eliminate the hematoma as much as possible. A drainage tube was inserted into the hematoma cavity to prevent residual hematoma. Retaining bone flap or not for the craniotomy group was depended on the judgement of the neurosurgeon according to the intraoperative situation.

#### MIPD group

According to the preoperative CT image, the puncture point was marked on the scalp and the puncture angle and catheter depth were planned. About 2% lidocaine was injected to anesthetize the skin around the puncture point. After the scalp was cut open with the puncture point as the center, the bone foramen was formed with an electric drill, and the stereotactic apparatus was installed and debugged. According to the puncture target, the hematoma was pricked by the used drainage tube according to the pre-designed direction and depth. After the bloody fluid flowed out, drainage gently with a syringe, the drainage tube was fixed and then connected with the drainage bag. After the patients of the MIPD group returned to the ward, they were injected with 10,000–20,000 units of urokinase/2–5 ml of saline solution to liquefy the hematoma for 1–2 times/day for 2–3 h.

### Postoperative care

The evacuation effect of hematoma was evaluated by brain CT every day after surgery. When the hematoma vanished or the remaining volume was < 10 ml, the drainage tube was closed. After 24 h, the drainage tube was removed if the vital signs of the patient were steady and no increased intracranial pressure were observed. Peripheral blood samples were collected for examination at 7 a.m. every day after surgery. Follow-ups in the outpatient department or by telephone were performed at the 30-day functional outcome after diagnosis. The functional status at 30-days was evaluated for prognostic outcomes. A modified Rankin Scale score (mRS) was used to assess the prognosis of patients.

### Outcomes

We retrospectively collected demographic and clinical information of all patients enrolled in the current study from electronic medical records, such as demographic characteristics, vital signs at admission, Glasgow Coma Scale (GCS) score at admission, medical history of hypertension and diabetes, lifestyle history of smoking and/or drinking, hematoma volume, hematoma location (supratentorial or infratentorial hematoma), surgical approach (craniotomy or MIPD), and clinical laboratory tests.

Clinical laboratory tests included absolute neutrophil count (ANC) and absolute lymphocyte count (ALC) at admission, within 48 h after surgery and at 3–7 days after surgery. The NLR was calculated by the ratio of ANC to ALC at different time points. The hematoma characteristics were recorded within 24 h after admission based on brain CT, such as hemorrhage location (supratentorial or infratentorial hemorrhage) and volume [calculated using the ABC/2 software (14)], intraventricular hemorrhage, and subarachnoid hemorrhage.

The appearance of functional independence was defined as favorable outcome (mRS <3), whereas dead or severely disabled were defined as a poor outcome (mRS ≥ 3) (15).

### Statistical analysis

The SPSS 25.0 (IBM Corporation, Armonk, New York, USA) and GraphPad Prism 9 (GraphPad Software, San Diego, California, USA) were used for statistical analysis and graph plotting, respectively. All continuous data are presented as median (interquartile range, IQR) and were analyzed using Student's *t*-test or the Mann–Whitney test, as appropriate. Categorical variables were expressed as frequency (percentage) and were analyzed using the chi-squared test. Baseline variables considered clinically relevant or showed a univariate relationship with the outcome (*P* <0.1) were included in a multivariate logistic regression to determine independent variables associated with a 30-day unfavorable outcome. The receiver operating characteristic analysis (ROC) was used to evaluate the threshold values for NLR in different surgical groups. The value of *p* <0.05 was considered as statistically significant.

## Results

In the current study, a total of 128 patients (88 men and 40 women) with a median age of 60 years (IQR: 50–67 years) were enrolled (Table 1). In the overall participants, the median time from onset to admission was 3 h (IQR: 2–8.75 h). The admission GCS score was 10 (IQR: 7–12). The hematoma volume was 35.9 ml (IQR: 26.0–48.5 ml). The proportion of 94.5% patients for the hematoma location was supratentorial. In addition, 57 (44.5%) patients with ICH were treated with craniotomy and 71 (55.5%) were treated with MIPD.

**Table 1:** Baseline characteristics related to the 30-day outcome in surgical patients with ICH.

**Characteristic**	**Total, (*n =* 128)**	**Favorable Outcome (*n =* 32)**	**Poor Outcome (*n =* 96)**	***P***
Demographics				
Male, *n* (%)	88(68.8%)	23(71.9%)	65(67.7%)	0.660
Age, IQR, *Y*	60(50.0–67.0)	52(47.3–66.5)	62.5(51.3–67)	0.056
Time from onset to admission, IQR, h	3(2.0–8.75)	3.5(2–8)	3(2–8.5)	0.958
Vital signs at admission				
Temperature, IQR, °C	36.6(36.4–36.8)	36.6(36.3–36.7)	36.6(36.4–36.8)	0.571
Heart rate, IQR, bpm	80(71–90)	80(70–92)	80(71–88)	0.643
Respiratory rate, IQR, bpm	20(18–21)	20(18–21)	20(18–21)	0.806
Systolic blood pressure, IQR, mmHg	169(151–191)	161(143–177)	172(153–198)	0.032*
Diastolic blood pressure, IQR, mmHg	99(89–110)	96(90–110)	99(88–110)	0.934
Admission GCS score, IQR,	10(7–12)	10.5(9–13)	9(7–11)	0.002*
Diabetes mellitus, *n* (%)	19(14.8%)	3(9.4%)	16(16.7%)	0.315
Hypertension, *n* (%)	95(74.2%)	22(68.8%)	73(76.0%)	0.414
Alcohol use, *n* (%)	29(22.7%)	8(25.0%)	21(21.9%)	0.715
Current smoking, *n* (%)	37(28.9%)	10(31.3%)	27(28.1%)	0.736
Hematoma volume, IQR, mL	35.9(26.0–48.5)	29.4(19.7–41.6)	36.3(29–63.4)	0.006*
Hematoma location				0.328
Supratentorial				
Lobar, *n* (%)	33(25.8%)	10(31.2%)	23(24.0%)	
Deep, *n* (%)	88(68.7%)	19(59.4%)	69(71.9%)	
Infratentorial				
Cerebellum, *n* (%)	7(5.5%)	3(9.4%)	4(4.1%)	
Intraventricular hemorrhage, *n* (%)	47(36.7%)	11(34.4%)	36(37.5%)	0.751
Surgical approach				0.010*
Craniotomy *n* (%)	57(44.5%)	8(25.0%)	49(51.0%)	
MIPD, *n* (%)	71(55.5%)	24(75.0%)	47(49.0%)	
NLR1, IQR	13.7(10.2–16.9)	8.8(7.3–13.0)	14.8(12.8–19.6)	0.001*

GCS, Glasgow coma scale; MIPD, minimally invasive puncture and drainage; NLR1, neutrophil-to-lymphocyte ratio within 48 h after surgery; IQR, interquartile range; n, number; *P < 0.05.

**Figure 1:**
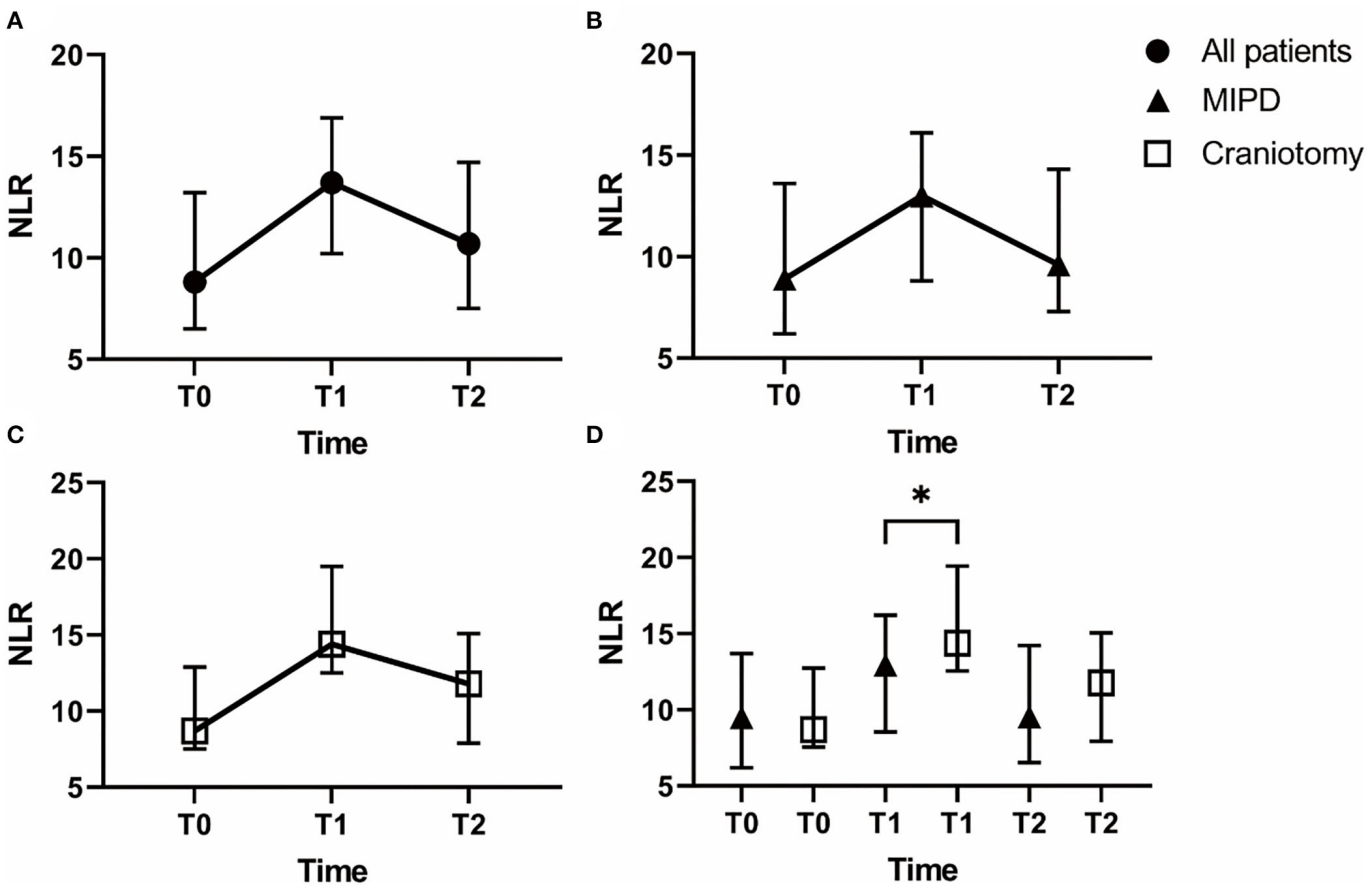
The median neutrophil-to-lymphocyte ratio (NLR) value over time between patients with a different surgical approach. The NLR of different time points in all patients **(A)**, in the MIPD group **(B)** and in the craniotomy group **(C)**. The difference of NLR between the MIPD group and the craniotomy group **(D)** (NLR, neutrophil-to-lymphocyte ratio; T0, at admission; T1, within 48 h after surgery; T2, at 3–7 days after surgery; * *p* < 0.05).

According to Figure 1A, the changing trend of NLR was to increase first and then decrease and peaked within 48 h after surgery (T1). When grouped by surgery (Figures 1B,C), the same trend emerged both in the MIPD group and the craniotomy group. NLR1 (NLR within 48 h after surgery) in the craniotomy group was higher than in the MIPD group (*p* < 0.05) (Figure 1D).

A univariate analysis showed that age, systolic blood pressure, admission GCS score, hematoma volume, surgical approach, and NLR1 were all risk factors for 30-day outcomes (Table 1). In the multivariate logistic analysis (Table 2), baseline variables included the risk factors considered clinically relevant (16–18) and the above significant characteristics. NLR1 was an independent risk factor of the 30-day outcomes in patients with ICH after surgical treatment.

**Table 2:** The multivariate logistic regression analysis of variables associated with the 30-day poor outcome.

**Characteristic**	**OR**	**95%CI**	***P***
Male	0.603	0.174–2.087	0.425
Age	1.049	0.999–1.102	0.057
Systolic blood pressure	1.012	0.992–1.033	0.243
Admission GCS score	0.866	0.683–1.096	0.231
Hematoma volume	1.025	0.984–1.068	0.242
Hematoma location	0.370	0.034–4.034	0.414
IVH	0.711	0.206–2.459	0.590
Surgical approach	1.816	0.538–6.344	0.329
NLR1	1.342	1.156–1.558	<0.001*

GCS, Glasgow coma scale; IVH, Intraventricular hemorrhage; NLR1, neutrophil-to-lymphocyte ratio within 48 h after surgery; OR, odds ratio; CI, confidence interval; *P < 0.05.

**Figure 2:**
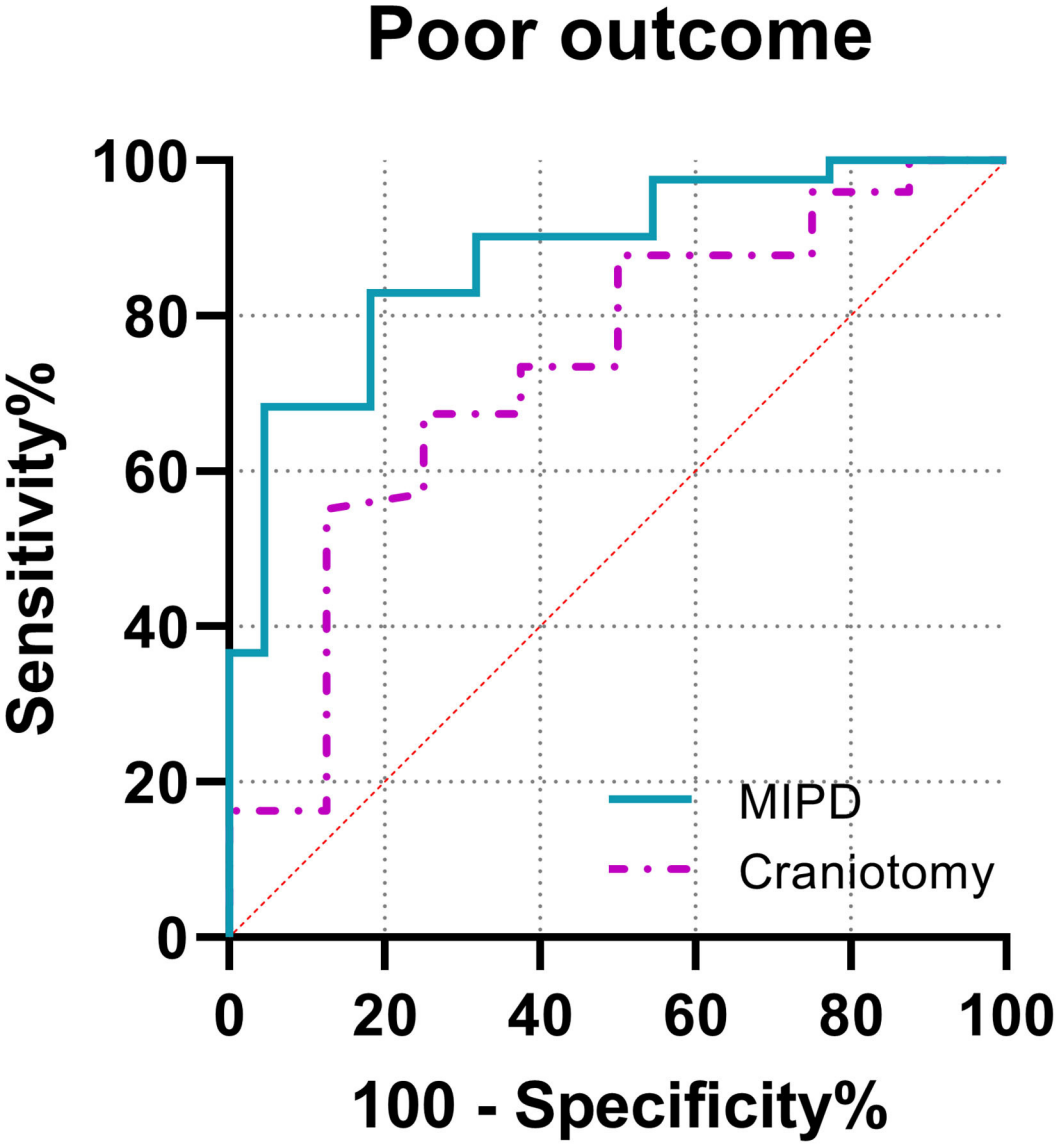
Receiver operating characteristic (ROC) curves of NLR1 in different surgery. The area under the curve (AUC) 0.877 (95% confidence interval [*CI*], 0.791–0.963; *p* < 0.0001) for NLR1 in the MIPD group; area under the curve 0.731 (95% *CI*, 0.539–0.923; *p* = 0.038) for NLR1 in the craniotomy group.

From the results of ROC (Figure 2), the best predictive cut-off value was 12.35 (sensitivity 82.9%, specificity 81.8%, positive predictive value, 4.55; and negative predictive value, 0.21) in the MIPD group and 14.46 (sensitivity 55.1%, specificity 87.5%, positive predictive value, 4.41; and negative predictive value, 0.51) in the craniotomy group.

## Discussion

In this study, the dynamic trend of NLR was to increase first and then decrease and peaked within 48 h after surgery. Moreover, NLR within 48 h after surgery was an independent risk factor of the 30-day outcomes in patients with ICH after surgical treatment. We also detected that the peak NLR >12.35 or 14.46 in patients receiving MIPD or craniotomy suggested a poor prognosis, respectively.

The inflammatory response starts immediately after hematoma formation in the brain. Microglia are stimulated by hematoma components and promote peripheral inflammatory cell infiltration through the release of pro-inflammatory cytokines and chemokines (19, 20). Neutrophils are the earliest subtypes of leukocytes that infiltrate the hemorrhagic brain by the blood-brain barrier (21). In a study of patients with ICH, the surrounding tissue of brain hematoma in patients confirmed that leukocyte infiltration occurred within 8 h after intracerebral hemorrhage and further increased within 1 day after onset (22). Another study showed that the number of neutrophils increased in peripheral blood may lead to central neutrophils infiltration, which may cause adverse outcomes (23). In addition, lymphocytes are apoptotic and deactivated by overactivation of the sympathetic nervous system and the hypothalamic-pituitary-adrenal axis in the immunodepression induced by stroke (24). Moreover, lymphocyte is a major component of the cellular immune system, and the decrease of lymphocyte is considered a marker of brain damage within 12 h after injury (24). Since inflammation is closely related to ICH pathophysiology, NLR, which reflects the information on both the innate and adaptive compartments of the immunity, may reasonably represent a surrogate biomarker of the likelihood of secondary brain injury and vulnerability to post-stroke complications (25). From the clinical perspective, although there was no evidence of infection, a significant proportion of patients with ICH in the acute stage is often observed to display an increase in peripheral white blood cells. At this moment, the local and systemic inflammatory response and the level of patients with acute ICH may be reflected by NLR (26).

The main finding of the present study was that the dynamic change of NLR in patients with ICH who underwent surgery was to increase first and then decrease, and reached a peak value within 48 h. A similar trend of NLR in patients with ICH undergoing surgery had not been reported in the pervious study, but was observed in patients with severe traumatic brain injury who underwent surgery (27). This change may be caused by many underlying factors. It is speculated that the process of the occurrence and progression of ICH, the inflammatory response was intense gradually, and the NLR value increased accordingly. As the disease improves, the inflammatory response fades away, and the NLR decreases accordingly (18, 27). In addition, surgical treatment may prevent hematoma expansion, block the release of inflammatory products from hematomas, and thus intervene the pathological processes after disease onset (11). Those may result in the peak NLR within 48 h after surgery. Considering secondary damage to brain tissue and the influence of inflammatory response caused by surgery (28), our study suggested that the NLR within 48 h after the surgery has more predictive value for the prognosis of patients with ICH who have undergone surgery than the NLR at other time points.

Our univariate analysis showed that the admission GCS score, hematoma volume, and NLR1 were correlated with 30-day outcomes of surgically treated patients. After multivariate logistic analysis, only NLR1 was found to be associated with prognosis. In previous studies, the admission GCS score and hematoma volume have been considered the predictors of prognosis in patients with ICH (16, 18). However, they are not completely satisfactory, and NLR may be used as a supplementary indicator. From a clinical perspective, the standardization for assessment of the admission GCS score is difficult to achieve and the accuracy of the GCS score is often affected by various factors, such as intubation (2). As for hematoma volume, it is not possible to follow-up frequently due to the exorbitant cost and radioactivity of CT. In comparison, NLR is cheaper and relatively easy to obtain from blood samples and more easily to observe dynamically. In addition, the peak NLR reflects the changes in inflammatory response in patients with ICH, which is helpful to timely adjust the treatment regimens. Therefore, NLR, especially the peak value of NLR, should be paid more attention during treatment. However, inconsistent with the previous study (12), the admission GCS score and hematoma volume were not associated with poor outcomes of patients with ICH in our study. The admission GCS score is highly subjective, which may be the reason for the different correlation between GCS score and prognosis in different studies. Since the hematoma volume is one of the bases to determine the surgical method, patients with different hematoma volumes have received appropriate treatment. Therefore, the influence of hematoma volume on prognosis may be relatively weak in this study.

In this study, the peak NLR >12.35 or 14.46 in patients treated with MIPD or craniotomy suggested a poor prognosis, respectively. The cut-off values in our study were similar to previous studies, which reported that range from 4.58 to 12.97 in previous studies (8, 13). The area under the curve (0.877 and 0.731) also suggested a satisfactory predictive power for the cut-off values of NLR in this study. Clinically, patients with ICH in the acute stage often need a dynamic routine blood examination, so it is convenient to obtain the peak value of NLR without additional costs. The treatment of patients with ICH at the acute stage is of great significance for prognosis, and the occurrence of peak NLR can help clinicians to judge the therapeutic effect of intervention measures, so as to formulate a more beneficial treatment plan for patients. Early measures to reduce the inflammatory response may be helpful to improve prognosis, such as therapeutic hypothermia (29). More than that, the current prognostic evaluation model of patients with ICH, such as ICH score, is mainly based on clinical information and lacks corresponding attention to laboratory biomarkers. Our study on the prognostic value of NLR provided new ideas for exploring new prognostic indicators with clinical significance (30).

Several limitations in the study should be taken into account. First, this was a retrospective study with a small sample. Second, the follow-up time was short. Third, although those known factors related to prognosis were concerned in the multivariate logistic regression analysis, other potential confounding factors might be ignored in the current study.

## Conclusion

The dynamic trend of the NLR of patients with ICH was to increase first and then decrease and peaked within 48 h after surgery. The peak NLR was an independent risk factor of the 30-day outcomes in patients with ICH after surgical treatment. The peak NLR >12.35 or 14.46 in patients receiving MIPD or craniotomy represented a poor prognosis, respectively. Further high-quality studies with large samples and multicenter are needed to verify the above results in the future.

## Data availability statement

The raw data supporting the conclusions of this article will be made available by the authors, without undue reservation.

## Ethics statement

The studies involving human participants were reviewed and approved by the Medical Ethics Review Committee of the First Affiliated Hospital of Chongqing Medical University and the Dianjiang People's Hospital of Chongqing. The Ethics Committee waived the requirement of written informed consent for participation.

## Author contributions

YZ: conceptualization, data curation, formal analysis, methodology, writing-original draft, writing, reviewing, and editing the manuscript. YX: data curation, formal analysis, methodology, resources, writing, reviewing, and editing the manuscript. SL: formal analysis, software, writing, reviewing, and editing the manuscript. MH: conceptualization, methodology, resources, supervision, validation, writing, reviewing, and editing the manuscript. All authors contributed to the article and approved the submitted version.

## Conflict of interest

The authors declare that the research was conducted in the absence of any commercial or financial relationships that could be construed as a potential conflict of interest.

## Publisher's note

All claims expressed in this article are solely those of the authors and do not necessarily represent those of their affiliated organizations, or those of the publisher, the editors and the reviewers. Any product that may be evaluated in this article, or claim that may be made by its manufacturer, is not guaranteed or endorsed by the publisher.

## Abbreviations

NLR: neutrophil-to-lymphocyte ratio
ICH: spontaneous intracerebral hemorrhage
MIPD: minimally invasive puncture and drainage
GCS: Glasgow Coma Scale
ANC: absolute neutrophil count
ALC: absolute lymphocyte count
CT: computed tomography scan
ROC: receiver operating characteristic analysis
mRS: modified Rankin Scale score
IQR: interquartile range.
